# First Case of Schizophrenia and OCD as Presentations of TK2-Related Mitochondrial DNA Depletion Myopathy: A Case Report

**DOI:** 10.1192/j.eurpsy.2026.11046

**Published:** 2026-07-31

**Authors:** S. H. Ngoh, A. Gudi

**Affiliations:** 1 MOH Holdings; 2 Psychiatry, Singapore General Hospital, Singapore, Singapore

## Abstract

**Introduction:**

Mitochondria generate energy in the form of adenosine triphosphate (ATP), integral in regulating cellular activities. Mutations in mitochondrial DNA occur when excess free radicals are produced during abnormal electron transport, causing cumulative oxidative stress and decline in mitochondrial function. The brain is particularly susceptible to oxidative damage due to its high oxygen consumption and low levels of antioxidant enzymes. This predisposes to neuropsychiatric manifestations, which may be exacerbated by psychotropics that further impair mitochondrial function.

**Objectives:**

To highlight clinical clues that distinguish primary psychiatric disorders from neuropsychiatric manifestations of mitochondrial disease, and to outline key prescribing principles in this context.

**Methods:**

Non-systematic review of the literature and report of a case study

**Results:**

We present Mdm K, a 59-year-old Chinese lady with papillary thyroid cancer, congenital alopecia and eczema, treated for schizophrenia and OCD for over 30 years before a diagnosis of possible TK2-related mitochondrial DNA depletion myopathy—the first reported case with neuropsychiatric features. Her psychiatric symptoms included contamination obsessions, cleaning compulsions, elementary auditory hallucinations, and negative symptoms, remaining stable on low-dose risperidone and fluoxetine without relapses. In 2023, she developed post-operative drowsiness and desaturation, with exam showing oculopharyngeal weakness and proximal myopathy. CK >10,000, aldolase 96.6, EMG with myopathic changes, and biopsy suggested mitochondrial myopathy. Genetic testing revealed two TK2 variants, also present in her sister. Despite months of psychotropic non-compliance, she reported only intermittent hallucinations without OCD relapse.

**Conclusions:**

Differentiating primary psychiatric disorders from neuropsychiatric symptoms of mitochondrial disease is crucial, as some psychotropics may worsen mitochondrial function. Literature reveals no clear guidelines and conflicting recommendations. Three predictors of organic pathology include: (a) multi-organ involvement, (b) cognitive impairment with negative symptoms, and (c) well-controlled symptoms on low-dose psychotropics. The absence of pharmacological guidelines highlights the need for research on psychotropic–mitochondrial interactions and potential adjunctive antioxidant or mitochondrial-targeted therapies. Meanwhile, safe prescribing should follow the principles of ‘start low, go slow’ with careful titration.

**Disclosure of Interest:**

None Declared

